# Low-Power IoT for Monitoring Unconnected Remote Areas

**DOI:** 10.3390/s23094481

**Published:** 2023-05-04

**Authors:** Alessandro Andreadis, Giovanni Giambene, Riccardo Zambon

**Affiliations:** Department of Information Engineering and Mathematics, University of Siena, Via Roma 56, 53100 Siena, Italy

**Keywords:** IoT, UAV, LoRa/LoRaWAN, opportunistic connectivity, energy efficiency, LPWAN

## Abstract

This paper deals with IoT devices deployed in remote areas without terrestrial Internet connectivity. We consider connecting IoT devices on the ground to the Internet through an aerial system based on an Unmanned Aerial Vehicle (UAV) for smart agriculture and environmental monitoring. The UAV flying over the remote area receives data from distributed IoT devices. The transmissions between the ground sensors and the UAV are carried out via LoRa. We have proposed a synchronization protocol for the opportunistic communication of LoRa IoT devices with a gateway onboard the UAV to save node battery life. Class A LoRa nodes on the ground transmit only when the UAV is expected to pass close to them; otherwise, they stay in the sleeping state most of the time. This paper provides a detailed description of the formulation of the synchronization protocol. The UAV’s flying dynamics have been considered for characterizing its speed and the time of visibility of each IoT sensor. Our model has allowed an analytical approach that can help to determine the best settings for LoRa transmissions. Finally, experiments have been carried out to assess the path loss attenuation, and a laboratory setup of the synchronization protocol has been implemented for the preliminary validation of our scheme.

## 1. Introduction

The increasing technical innovations of the last few years have allowed new opportunities and applications for the Internet of Things (IoT), and the advent of Low-Power Wide-Area Network (LPWAN) technologies has facilitated massive access to IoT nodes [1]. The IoT is becoming extremely popular in agriculture and environmental monitoring, where smart agriculture applications can improve sustainability and give farms an economic return of up to 25% of the harvest. For this reason, nowadays, many sensors are used for smart agriculture applications, and the trend is increasing. For instance, moisture sensor monitoring is particularly useful in understanding irrigation needs: when soil moisture is below a certain level, water can be provided through the activation of local irrigation actuators connected with sensor nodes, allowing for more efficient irrigation and greater sustainability and reducing human intervention.

When IoT devices are deployed in remote and rural areas, a significant criticality is the lack of Internet connectivity and local power supply. This issue can be overcome by adopting low-power, long-range radio communications using an IoT Gateway (GW) onboard an aerial node, a so-called Unmanned Aerial Vehicle (UAV). The UAV is envisaged to periodically pass over the IoT sensors’ area in order to collect data measurements and to forward them to the Internet.

Although there are several LPWAN technologies, in this study, we focus on LoRa because this low-power communication technology can allow devices to communicate with other nodes kilometers away, lasting several years without battery replacements. Furthermore, LoRa allows work to be conducted on an open-source Medium Access Control (MAC) layer (specified as LoRaWAN) to implement new protocol solutions or other specific scenario-based upgrades.

This paper deals with the communication of ground IoT nodes with a GW placed onboard a UAV passing in their proximity. This work also proposes an opportunistic protocol to synchronize the ground node transmission with the UAV pass, thus enabling the node to stay in the sleeping state most of the time and save energy. This solution can coordinate node transmissions in order to avoid collisions, a key feature in the perspective of a future massive deployment of sensor nodes. This work includes a study on propagation to assess the path loss attenuation when sensors are close to the ground. Moreover, an analytical model has allowed us to identify the best physical layer settings of the LoRa for the opportunistic scheme in terms of the probability that a sensor can communicate with the UAV during its visibility interval. This synchronization protocol has also been tested in the laboratory.

The present work extends the study of the same authors in [2] by including significant improvements as follows: (i) the UAV flight dynamics and wind conditions have been considered; (ii) an analytical approach is developed to evaluate the impact of synchronization; and (iii) a preliminary laboratory setup has been presented to validate our scheme with LoRa devices.

This paper is organized as follows. Section 2 introduces the LoRa/LoRaWAN technology, describing the use of UAVs in LoRa systems and providing a literature survey. Our UAV-based system is then described in Section 3, while Section 4 deals with the opportunistic protocol designed to manage the communication between ground nodes and the UAV. Section 5 analyzes the probability that the sensors can communicate with the UAV passing over them. Experimental results regarding path loss attenuation for a rural area and test validation of the opportunistic protocol are presented in Section 6, and discussion on the achieved results, lessons learned, and open issues are provided in Section 7. Finally, Section 8 outlines the concluding remarks. 

## 2. Background

This section provides an overview of enabling communication technologies (primarily focused on LPWAN solutions) and systems for mobile coverage of remote areas based on UAVs. Furthermore, it reports state-of-the-art solutions for opportunistic communications between on-field sensor nodes and moving GWs.

### 2.1. Selection of LPWAN Communication Technology

The Third Generation Partnership Project (3GPP) solution for IoT radio communications is based on the NB-IoT, recently included in Release 17 for Non-Terrestrial Networks (NTN) [3]. The NB-IoT uses a subset of the well-known Long-Term Evolution (LTE) technological features while limiting its operation to a single carrier bandwidth of 180 kHz (narrowband technology). By applying the NB-IoT to our scenario, a cell of coverage has to be irradiated by the UAV, similarly to a base station. IoT nodes and the GW can transmit power levels up to 20 dBm or more. Although the NB-IoT serves higher-value IoT markets interested in very low latency and high quality of service, it is not open source, and its deployment is limited by base station coverage, making it unsuitable for remote regions [1]. Similar considerations apply to LTE-cat M technology, which still relies on cellular base stations; moreover, in case of congestion, the Cat-M IoT device can be forced to disconnect from the network and be allowed to re-attach at a more favorable time. As an alternative, Sigfox provides terrestrial cellular-like coverage for IoT applications, but this system requires its proprietary network. An entirely open solution can be offered by weightless wireless technology, running in the unused UHF sub-GHz TV unlicensed spectrum; however, it has not achieved great popularity and few hardware implementations are available.

In this work, we adopt the long-range and low-power technology provided by LoRa with a LoRaWAN open source MAC layer.

### 2.2. LoRa/LoRaWAN

LoRa is the physical layer of a proprietary wireless IoT technology specifically intended for stable radio connectivity [4]. A LoRa device can reach a maximum range of 5 km for urban and 20 km for rural areas under ideal propagation conditions. This range can be significantly reduced in the presence of dense vegetation. LoRa operates in the unlicensed Industrial, Scientific, and Medical (ISM) bands. Diverse frequency bands are available in different regions, such as 863–870 MHz in Europe (EU868), 902–928 MHz in North America (US915), 470–510 MHz in China, and 920–925 MHz in Korea. LoRa adopts a Chirp Spread-Spectrum (CSS) modulation that uses bandwidths of 125 kHz, 250 kHz, and 500 kHz (other options are possible). In Europe, the EU868 band supports a maximum of 16 channels of 125 kHz. It can achieve a maximum bit rate of 50 kbit/s. Transmissions can occur using different Spreading Factors (SF) and data transmission rates, where a lower *SF* value corresponds to a higher data rate. For a reference bandwidth of 125 kHz, the bit rate ranges from 5.47 kbit/s for *SF* = 7 and Coding Rate (CR) = 1 to 183 bit/s for *SF* = 12 and *CR* = 4. The *SF* values also determine different coverage ranges due to different receiver sensitivity levels (e.g., from −123 dBm with *SF* = 7 to −137 dBm with *SF* = 12, using a 125 kHz bandwidth for our LoRa transceiver, as explained in Section 6); adopting a lower *SF* value implies a shorter coverage range.

According to the requirements of the “Conférence Européenne des administrations des Postes et des Télécommunications” (CEPT), in the EU868 band, the transmission duty cycle for end-devices cannot be larger than 1% and the maximum transmission Effective Radiated Power (ERP) is *P_t_* = 25 mW (14 dBm) [5]. The ERP is measured using a half-wave dipole (with antenna gain at most equal to 2.15 dBi) instead of an isotropic antenna, as in the Effective Isotropic Radiated Power (EIRP). Then, the following formula holds: *EIRP* = *ERP* + 2.15 dBm.

Semtech has patented LoRa chips supporting the implementation of the physical layer [6]. These transceivers are characterized by good sensitivity that covers long ranges within the ERP power constraints. The LoRa PHY chain includes encoding, whitening, interleaving and Gray mapping. Modulation adds a Forward Error Correction (FEC) in every data transmission based on a simple binary Hamming code. 

Moreover, the LoRa Alliance has defined the LoRaWAN protocol, specifying the packet format and an Aloha-like protocol. In particular, LoRaWAN differentiates the devices into Classes A, B, and C. Class A devices sleep most of the time and can transmit at any time, depending on their needs; they wake up to send their up-link messages, then they start to listen after a programmed waiting time for two predefined consecutive receive windows. In Class B, the response uses extra receive windows regularly scheduled via a GW beacon signal; in Class C, the feedback can be received at any time apart from the transmission time [7]. Class A allows for the lowest power consumption.

**Figure 1:**
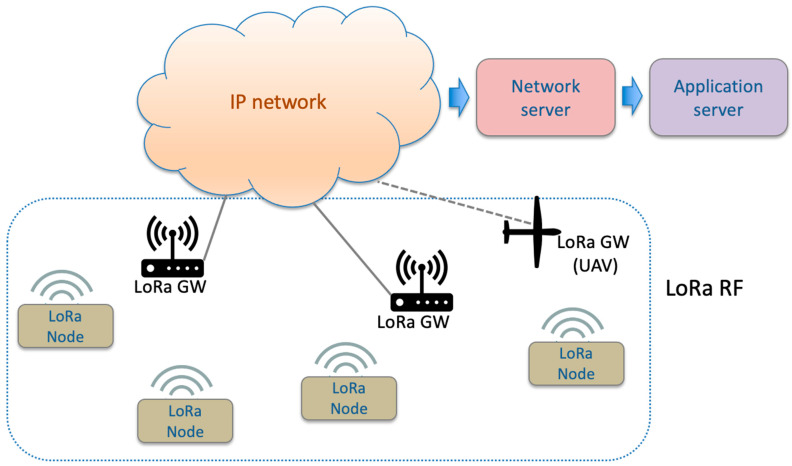
LoRa/LoRaWAN communication system and architecture.

The LoRaWAN architecture envisages a network of LoRa nodes with sensors, a LoRa GW, a network server, and an application server. A LoRaWAN network can be a private LoRaWAN network set for a specific scenario such as ours; alternatively, it can use a dedicated network such as The Things Network (TTN), which imposes a duty cycle restriction (‘fair access policy’): LoRa nodes can transmit for a maximum of 30 s per day. The star topology and the main components of the network envisaged by LoRaWAN are shown in Figure 1 [8].

One of the critical issues when using a UAV to collect data measurements from sensors on the ground is that UAV connectivity is opportunistic. Because of the movement of the UAV, it is not always available to nodes. Therefore, an opportunistic protocol needs to be carefully designed to coordinate the sensor nodes’ transmissions with the UAV passing by, thus allowing for energy-saving operations and longer duration of LoRa IoT devices’ batteries (an important requirement is a 5–10-year lifetime without replacing sensor batteries). We will address the opportunistic connectivity scheme for LoRa/LoRaWAN in Section 4.

### 2.3. Use of UAVs and Communications with IoT Nodes

**Figure 2:**
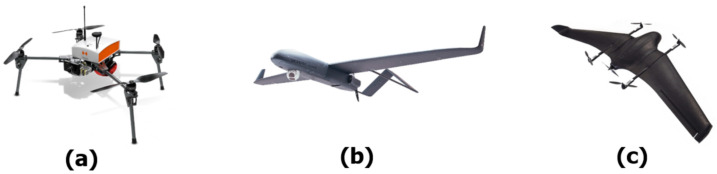
Different UAV types: (**a**) rotary-wing, (**b**) fixed-wing, (**c**) hybrid case.

In 3GPP, there is great momentum nowadays on NTN as a component of 5G systems and beyond. Not only UAVs but also High-Altitude Platform (HAP) stations (18–21 km of altitude) and Low-Earth Orbit (LEO) satellites (500–2000 km altitude) are valid solutions to communicate with ground IoT sensor nodes [9,10]. Here, we focus on using UAVs since they can be flexibly deployed where needed and represent cheaper solutions. In addition, they stay closer to the ground, thus facilitating the link budget with low-power IoT nodes on the field. Consequently, the system is more energy efficient and increases the battery lifetime of the IoT nodes [11]. UAVs can be classified according to three categories with different features, as shown in Figure 2. Rotary-wing UAVs are equipped with vertical lift rotors, allowing them to hover in the air. They are ideal for collecting videos and images or distributing treatments in smart agriculture. Fixed-wing UAVs are similar to common airplanes; they need a launch pad for take-off. Fixed-wing UAVs allow for reduced battery consumption and cover longer distances. The third category, hybrid UAVs, is a mix of fixed- and rotary-wing UAVs, having the ability to switch between the two flying modes.

Several modules are necessary inside a UAV: propeller(s), engine(s), flight control board, radio transmitter and receiver modules, a rechargeable battery or a tank in the case of a fuel engine, and a Global Navigation Satellite Systems (GNSS) module [12]. The UAV can be programmed to scan a specific area for agriculture and environmental monitoring applications [13] through a waypoint-based pre-determined path. The acquired measurements from sensors can be communicated to the LoRa server by adopting different technologies: with a 5G terrestrial gNodeB covering the scanned areas, through satellite communications, or by storing the data in the UAV memory and downloading them when the UAV returns to the starting point for landing/battery recharging.

### 2.4. Literature Survey

There is common use in the literature of LoRa transceivers for IoT applications, as in [14], which provides a comprehensive review of practices and strategies for sustainable and energy-efficient IoT technologies.

The work in [15] investigates current practices, challenges, and policies related to IoT network implementation. It discusses power consumption related to data centers, sensor nodes, and machine-to-machine communications, thus evaluating the threat of e-waste to the environment.

Reference [16] introduces a novel energy-efficient routing criterion for a smart grid environment. It adopts LoRa nodes implementing optimal channel and path selection techniques, thus obtaining a lower energy consumption than other routing protocols such as Low-Energy Adaptive Clustering Hierarchy (LEACH), Stable Election Protocol (SEP), and Distributed Energy Efficient Clustering (DEEC).

In [17], different WiFi-based sensor network configurations are examined for soil monitoring to identify the effects of the rural environment on the signal; propagation tests were performed which adopted three different types of testbeds (i.e., a grass field with low vegetation and regular terrain, a thicket with irregularities, a field of citrus) with on-ground, near-ground, and above-ground receiver placements. The maximum theoretical coverage was obtained for each configuration, showing that vegetation introduces high variability, especially in the presence of high foliage density.

The work in [18] proposes a LoRa sensor network for monitoring water quality in coastal areas. The adopted approach has been tested in a real environment to check the operativity of the sensors and architecture. However, this work does not implement scenarios involving real UAVs, but introduces a related opportunistic protocol and on field measurements related to an UAV emulated scenario.

The paper in [19] uses a LoRa transceiver of the SX1276 type. There is mobility between the end node (sensor) and LoRa GW. The authors adapted an existing open source firmware, called SX1276 Generic Ping-Pong, so the GW can send a customized beacon to advise about its reachability. These authors use *SF* = 12 and consider a 250 m connectivity radius with a transmission power of 14 dBm in the presence of vegetation. The approach proposed in this work implies that the sensor node transceiver stays awake to receive the ping message from the occasional pass of the GW. This approach is inefficient and could drain the node battery quickly.

Reference [20] proposes an opportunistic multi-hop routing protocol for LoRa-based intermittently connected networks called LoRaOpp. This solution envisages various setup scenarios but is not focused on using GWs on UAVs. This paper considers multi-hop data exchange between nodes and between the nodes and the GW, including the use of several techniques to minimize the number of forwarded packets in the network.

The work in [21] presents a data collection platform for smart cities that envisages mobile and fixed nodes equipped with WiFi and LoRa interfaces to forward packets in intermittently connected networks. This platform involves node sensors, sink stations connected to a fixed network, and mobile devices functioning as relays between nodes and sink stations. Specifically, this platform employs an alternative LoRa MAC protocol that leverages the presence of multiple sink stations in the network.

A scheme to coordinate the sensor transmission with the pass of the flying GW is to use the Wake-up Radio (WuR) technology [22]. WuR is a secondary always-on Ultra-Low Power (ULP) radio subsystem connected to the main node. The WuR continuously listens to the channel while the main radio is in sleeping mode. When a specific signal called a Wake-Up Beacon (WUB) is received, the WuR wakes up the main radio through an interruption. Thus, the WuR approach allows for an asynchronous wake-up of the main node with low latency. As LoRa and WuR present orthogonal features (long range with LoRa and short range with WuR), recent works have proposed combining these technologies on the same device to achieve energy efficiency and reduce latency. Typically, only the ULP WuR is always-on and listening to the channel, while the other components are in power-saving (sleep) mode. With this approach, the WuR range is very limited (about 120 m): in our case, the UAV should fly closer to the ground so that the nodes can receive its WUB. This constraint poses some limitations because it is more efficient to have the UAV flying at higher altitudes since the propagation conditions are better, and the UAV can cover a larger area with longer visibility intervals.

The paper in [23] adopts a rotary-wing UAV for a forest monitoring system based on LoRa. Each ground node hosts multiple sensors (temperature, light, and humidity). The LoRa GW is hosted on a UAV, which is connected to the network via WiFi or 4G systems, and it is expected to hover and collect data from the sensors of the covered area. The sensor node is always active in receiving mode and performs a network join procedure and a signaling handshake to transmit the data each time the UAV covers it. The node recognizes the arrival of the UAV that sends a beacon signal. Our system adopts a more efficient approach, where the LoRa node is in the sleeping state for most of the time, keeping all the benefits of the low-power LoRa transmissions also in case of communication with a UAV. 

The main contributions of the works discussed above are summarized in Table 1.

## 3. System Description

**Figure 3:**
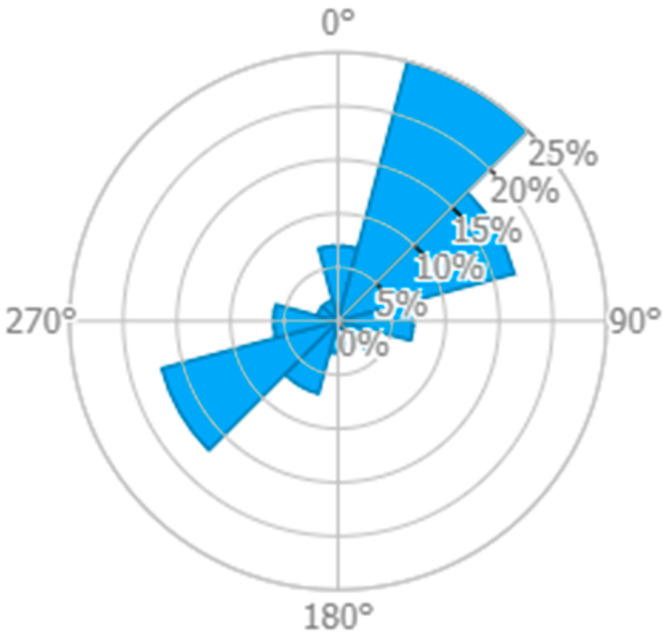
The wind rose for the selected location.

The operating context refers to a rural/remote area without network connectivity to support an IoT network, where data transmission is obtained through the adoption of a LoRa GW onboard a flying UAV. Specifically, the objective is to monitor a rural area along a river or other ‘linear’ infrastructures (e.g., pipelines), assuming the use of a fixed-wing UAV to perform this task, as there is no need for hovering functions, only to scan a specific area in the shortest possible time. LoRa sensors on the ground send periodic measurements. We have selected a reference location in Tuscany (close to Florence) and determined the wind speed and direction statistics at 120 m of altitude to be suitable for UAV flight. Data have been taken from the Wind Atlas [24] and are shown in Figure 3 below.

**Figure 4:**
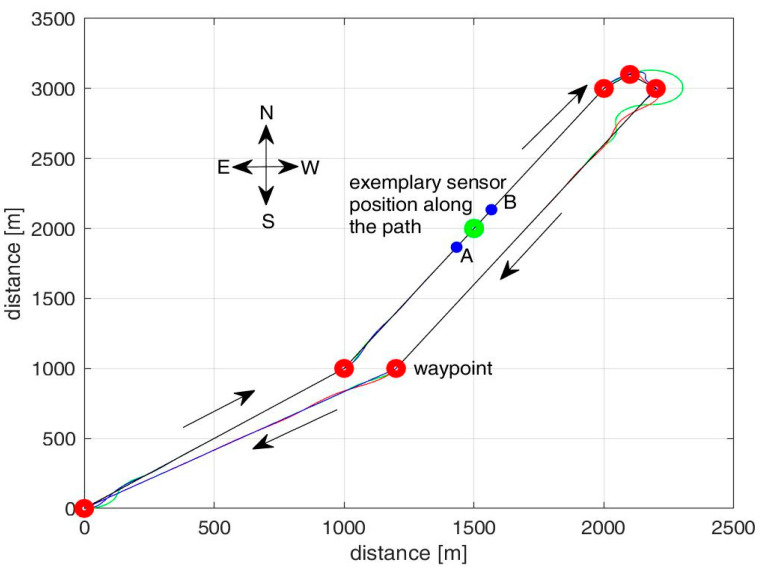
Envisaged UAV paths following the same waypoints with different wind conditions corresponding to distinct colors. The UAV nominal speed is *v_d_* = 70 km/h.

For our reference location, we have selected the two strongest wind conditions and neglected all the other cases for which we refer to the ideal condition of no winds. Then, according to Figure 3, we have the following wind statistics:

- Case 1: Average wind speed of 6.5 m/s from NNE for 25% of the time (UAV path shown in red in Figure 4);
- Case 2: Average wind speed of 6.5 m/s from WSW for 16% of the time (UAV path shown in blue in Figure 4);
- Case 3: Negligible wind speed for 59% of the time (UAV path shown in green in Figure 4).

The UAV route is predefined to follow waypoints. The UAV flight is controlled by an autopilot (Guidance, Navigation and Control system, GN&C) that is reproduced in our study by the UAV Toolbox provided by Matlab (fixed-wing case) [25,26]. This tool allows the simulation of the UAV, considering the flight dynamics and the effects of winds. In its flight, the UAV visits each waypoint with a tolerance of about ±50 m [27]. Figure 4 shows our UAV path based on waypoints and considering the three wind cases of our reference scenario and the UAV having to monitor the IoT sensors distributed along a river. If the sensors are deployed more sparsely, a UAV path optimization algorithm has to be adopted to select the shortest path to reach all of them; this is not the present case, since the sensors are deployed along a river modeled by linear segments. The presence of the winds modifies the actual UAV speed even if the controller onboard counteracts their effects and is set to a nominal UAV speed denoted as *v_d_* = 70 km/h. The results obtained from the Matlab UAV Toolbox simulator for the time needed to cover our path are 426 s, 422 s, and 398 s for the red, blue, and green wind cases, respectively. 

Let us refer to the position of an exemplary sensor along the UAV path, as shown in Figure 4. The UAV will start to be visible for this sensor when it reaches position A and ends its visibility interval at point B. The distance from A to B is 2*R*, where *R* is the coverage radius for the sensor node. The wind conditions affect the time when the UAV is at the minimum distance from the *i*-th sensor, *T_c_*_,*i*_, and the interval for which the UAV is visible to the sensor, Δ*_v_*(*SF*), where *SF* is the adopted spreading factor, *SF* Î {7, 8, 9, 10, 11, 12}. In nominal conditions without winds, we have:

(1) $$\Delta_{v}(SF)=\frac{2\sqrt{{R(SF)}^{2}-x^{2}}}{v_{d}},$$

where *x* denotes the distance of the sensor node position from the projection of the UAV path on the ground. The coverage *R* = *R*(*SF*) will be characterized later in this section based on a LoRa link budget.

The UAV can receive the transmissions of a sensor if it is within the coverage radius *R*(*SF*) and inside its UAV visibility interval Δ*_v_*(*SF*) (1). 

Let us recall that *T_c_*_,*i*_ denotes the time when the UAV reaches the closest point along its path from the *i*-th sensor node; this time is at the center of the UAV visibility interval for the *i*-th node. Table 2 shows the central visibility time *T_c_*_,*i*_ and the actual UAV speed *v* for the exemplary sensor in Figure 4; the impact of the wind conditions on *T_c_*_,*i*_ and *v* is evident.

**Figure 5:**
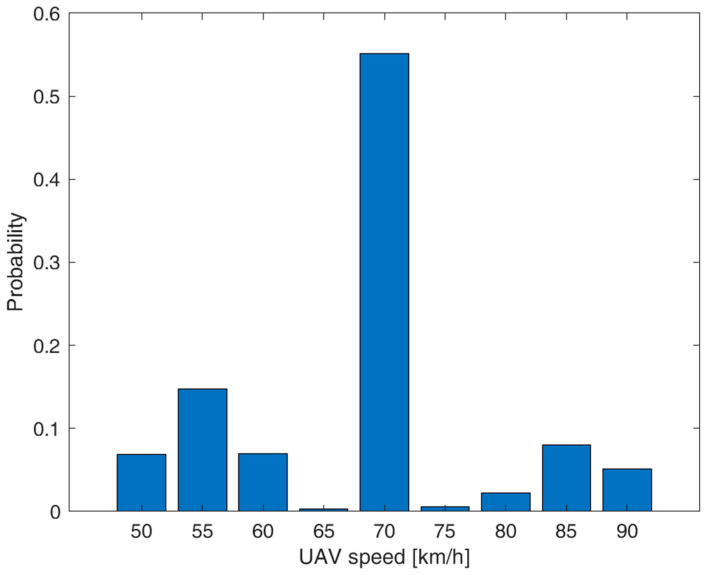
UAV speed distribution along the UAV path considering the wind conditions for our reference area and nominal UAV cruise speed *v_d_* = 70 km/h.

Referring to our scenario in Figure 4, Figure 5 shows the distribution (histogram) of the resulting UAV speed *v* as a result of the nominal cruise speed of 70 km/h and the effects of our wind conditions along the path. We can notice the peak caused by the nominal cruise speed of 70 km/h when there are no winds (case 3 above), with a probability of 59%. The effects of the winds in cases 1 and 2 cause an alteration in the UAV’s speed, and thus the UAV speed values range from 50 to 90 km/h. In our theoretical study, we will model the UAV speed’s probability density function *f_v_*(*v*) as a combination of a uniform distribution from *v_min_* = 50 to *v_max_* = 90 km/h and a discrete value at the nominal speed *v_d_* of 70 km/h as follows:

(2) $$f_{v}\left(v\right)=0.59\;\delta \left(v-v_{d}\right)+0.01025\;rect\left(\frac{v-v_{d}}{40}\right),$$

where *v* is the UAV’s effective flight speed in km/h, $\delta \left(v\right)$ is the Delta Dirac function centered at 0, and *rect*(*v*) is the unitary rectangular function from −0.5 to 0.5. Note that the term $\delta \left(v-v_{d}\right)$ in (1) is related to the nominal UAV speed *v_d_* corresponding to case 3 with a probability of 59%.

The signal to and from the UAV is affected by many scattering obstacles (e.g., buildings, trees). The signal received is the composition of many waves, including Line of Sight (LoS), reflected path, diffracted path, and scattering. These effects depend on the environment, the carrier frequency, and the UAV’s altitude. All these elements (commonly identified as clutter terms) cause the path loss attenuation to deviate from the free space or the two-ray models [28]. Even if the path loss model for our specific rural area case is studied in Section 6, we prefer to adopt a more general path loss model based on the work in [29], including an additional attenuation due to trees and foliage of 10 dB [30]. The resulting long-distance (large time-scale) path loss model depends on the distance *d* as follows:

(3) $$L\left(d\right)=L\left(d_{0}\right)+10\gamma {log}_{10}\left(\frac{d}{d_{0}}\right)+L_{veg}[dB],$$

where, for LoS conditions, we consider $L\left(d_{0}\right)$ = 116 dB for $d_{0}$ = 1 km, γ = 3, and $L_{veg}$ = 10 dB. In non-LoS conditions, we should consider an additional path loss attenuation term of 10–20 dB.

We refer to a UAV flying at an altitude *H* = 120 m (the limit value for the open flight category by the European Union Aviation Safety Agency, EASA [31]) and a GW receiver (LoRa SX 1276) with sensitivity *S* = {−124, −127, −130, −133, −135, −137} dBm for *SF* ∈ {7, 8, 9, 10, 11, 12}, respectively, with a 125 kHz bandwidth [32]. We consider omnidirectional antennas on both the transmitter and receiver (antenna gains *G_tx_* = *G_rx_* = 0 in dBi) with connector and cabling losses *L_con_* = 2 dB. Let *P_t_* denote the sensor transmission power. We consider connectivity to be achieved if the following condition is met:

(4) $$P_{t}+G_{tx}+G_{rx}-L\left(d\right)-L_{con}-M\geq S\left(SF\right),$$

where *M* is a margin to protect the transmission from typical shadowing effects caused by obstacles along the path from the transmitter and receiver; we typically assume *M* = 10 dB for a safe link budget.

The coverage radius *R* is the maximum value of *d* satisfying the condition in (4) that the received power is above the sensitivity threshold for the corresponding *SF*:

(5) $$R\left(SF\right)=10^{\frac{P_{t}+G_{tx}+G_{rx}-L\left(d_{0}\right)-L_{veg}-L_{con}-M-S\left(SF\right)}{10\gamma }}.$$

Note that we compute *R* in (5) referring to the uplink transmission (i.e., from sensors to the UAV), but we also apply it to the downlink, assuming that the uplink case is more critical.

**Figure 6:**
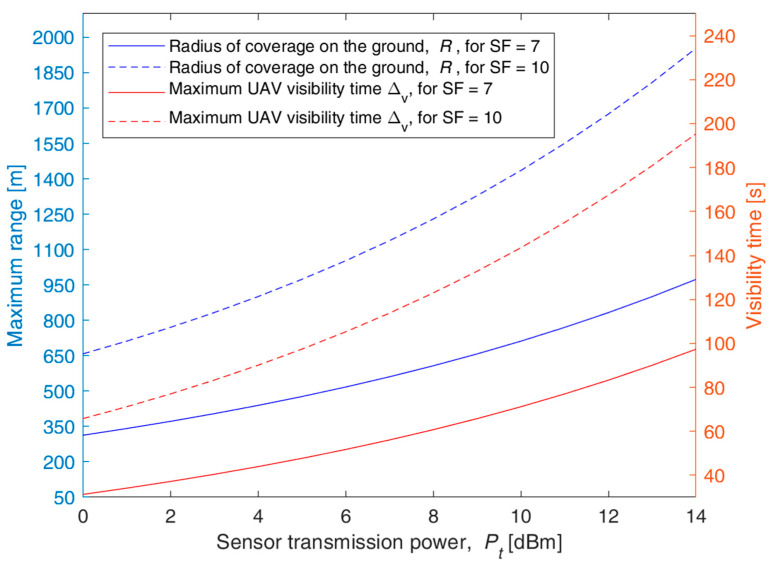
Sensor node radius of coverage *R*(*SF*) and maximum UAV visibility time Δ*_v_*(*SF*) versus the transmission power *P_t_* for *SF* = 7 and 10, bandwidth of 125 kHz, UAV altitude *H* = 120 m, and UAV nominal speed *v_d_* = 70 km/h.

**Figure 7:**
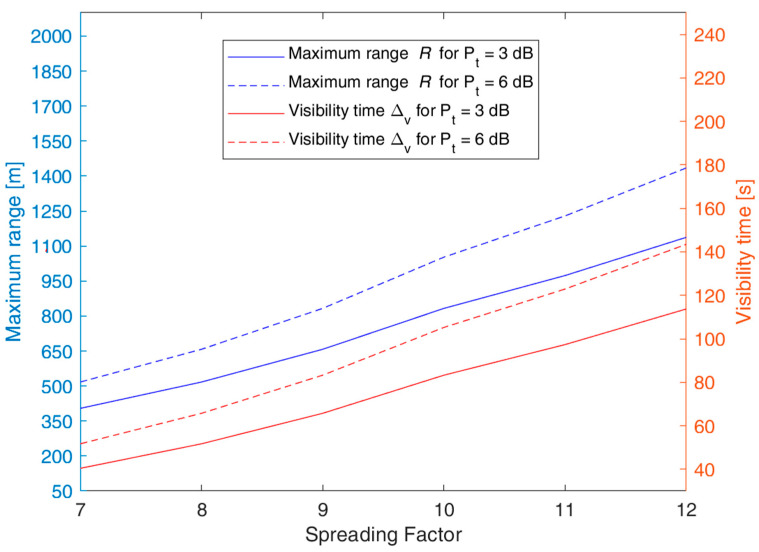
Sensor node radius of coverage *R*(*SF*) and maximum UAV visibility time Δ*_v_*(*SF*) versus the spreading factor *SF* for *P_t_* = 3 and 6 dB, bandwidth of 125 kHz, UAV altitude *H* = 120 m, and UAV nominal speed *v_d_* = 70 km/h.

Figure 6 shows the maximum range *R*(*SF*) and the corresponding maximum Δ*_v_*(*SF*) for *x* = 0 as functions of the sensor transmission power *P_t_* in dBm for *SF* = 7 and 10. Figure 7 provides a similar graph but having *R*(*SF*) and Δ*_v_*(*SF*) as functions of *SF* for sensor transmission power *P_t_* = 3 and 6 dB. For instance, with *P_t_* = 6 dBm, we obtain *R* = 500 m for *SF* = 7 and *R* = 1100 m for *SF* = 10. Correspondingly, the maximum UAV visibility time for a sensor on the ground is Δ*_v_* = 55 s for *SF* = 7 and 125 s for *SF* = 10 with nominal UAV speed conditions. The radius *R* and the visibility interval Δ*_v_* can be improved by increasing the *SF* value and/or the transmission power level *P_t_*. 

To complete the signal propagation characterization, we consider the probability of LoS for the communication between ground nodes and the UAV, *P_LoS_*. This probability is essential because the path loss model in (3) can only be applied in LoS conditions. According to [33], *P_LoS_* can be expressed by a sigmoid-type function as follows:

(6) $$P_{LoS}\left(\vartheta \right)=\frac{1}{1+ae^{-b\left(\vartheta -a\right)}},$$

where ϑ is the elevation angle in degrees of the communication between the ground sensor node and the UAV, and *a* and *b* are coefficients (we consider a = 5 and b = 0.3 for suburban and rural areas [33]). In our study, the value of ϑ for a sensor depends on the relative position of the UAV that changes with time because the UAV moves along its path. An example of the derivation ϑ for our case will be provided in Section 5.

## 4. Synchronization Protocol for Opportunistic Communications between GW and IoT Nodes

This section describes the proposed synchronization protocol that enables LoRa devices to communicate opportunistically with the GW on the UAV to increase energy efficiency. A key point to implement reliable communications between the flying GW (onboard the UAV) and end nodes is the capability for the end nodes to be in sending/receiving mode when the UAV is within the communication range. This task could be easily achieved by implementing a LoRa Class C node, which is always on, so it can transmit and receive information at any instant. However, this class of devices is intended for actuators or power-supplied sensors due to their high energy consumption [7]. One of the key innovations of this work is the adoption of LoRa Class A sensors for this task, allowing a very low power consumption (for instance, from 30 to 220 times lower than Class C sensors with *SF* = 7 [7]). To reach this goal, we envisage simple LoRaWAN modifications to support the synchronization of the ground node transmissions with the UAV pass time; in particular, we propose an opportunistic protocol that enables node transmission only when needed and keeps the sensor node in the sleeping state for the rest of the time to save battery energy. 

In the following, the description of the opportunistic protocol is provided. We are considering a single-channel GW on the UAV. We assume all the sensors transmit using the same LoRa *SF* value. The node position can be mapped during the deployment phase. Then, the node’s first transmission timing *T_0s_*_,*i*_ (corresponding to the time when the UAV is expected to reach the *i*-th sensor according to the first planned flight route with nominal speed *v_d_*) can be calculated using the Matlab UAV toolbox (Section 3) and pre-set in this sensor node. The node location is determined at the time of installation without needing a GPS module on the node (sensors are deployed in fixed locations). However, in the case of moving sensors (i.e., livestock monitoring, etc.), each node could also be equipped with a GPS module at the cost of extra energy consumption. The initial (i.e., “first round”) transmission timing *T_0s_*_,*i*_ is initialized in the node firmware to schedule its first transmission in the middle of the related UAV coverage time interval Δ*_v_*_,*i*_, thus achieving better margins if some variations from nominal conditions occur. In this way, the probability of LoS at the transmission time is maximized because the sensor transmits when the UAV is visible with the maximum elevation angle.

Our system is based on a route management algorithm composed of route planning and a UAV flight model. It defines the route and the expected transmission time for each sensor node to keep the synchronism with the next UAV pass. In this work, we are not interested in studying route optimization schemes. We consider the nodes along a river modeled by linear segments (we set the waypoints accordingly). A further study of route optimization is beyond the scope of the present paper, which is concerned with investigating the synchronization of sensor transmissions with the UAV pass. This route management algorithm is carried out based on the knowledge of sensor nodes’ positions and uses a UAV flight simulator (Matlab UAV Toolbox, fixed-wing case) to take the effects of wind into account. 

**Figure 8:**
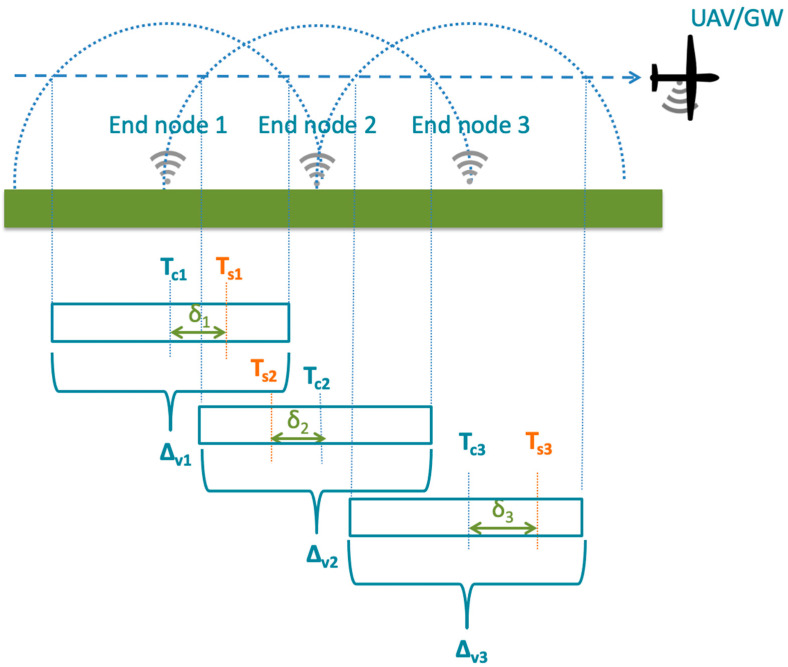
Planned transmission times, actual transmission times, and visibility intervals of sensor nodes.

The UAV communicating with the sensor nodes expects to receive the measurements from node *I* at time *T_c_*_,*i*_. The ideal condition for the protocol is that the *i*-th sensor transmission time *T_s_*_,*i*_ coincides with *T_c_*_,*i*_. However, we can lose this synchronism because of the following causes (see Figure 8):

1. A shift in the UAV’s departure time;
2. Coarse clock precision (clock drifts on sensors);
3. Alteration in the UAV’s flight speed with respect to the expected one because of wind variations;
4. The UAV’s autopilot precision and capability to reach the waypoints also in relation to the wind conditions.

- Hence, a synchronization scheme is envisaged where the UAV upgrades the *i*-th sensor transmission time at each pass. This is made possible using the ACK message as a reply to the measurement received by the UAV. The ACK message piggybacks the *D_i_* value for the sensor that represents an estimation of the time the sensor has to sleep before transmitting again for the next UAV pass; see the following Equation (7). The sensor receiving the ACK can then command its transceiver to enter a sleeping mode for time Δ*_i_* to save energy. By adopting this protocol, a node remains in the sleeping state most of the time. The device wakes up just for the time needed to take a measurement from the field and transmit it when the UAV is expected to pass close to it.

The adopted scheme with synchronism is *robust* since the sensor transmission has a tolerance made possible by the UAV’s visibility interval for the sensor with a duration Δ*_vi_* (*SF*) according to (1). Therefore, the interval is long enough that the sensor transmission falls inside it to communicate with the UAV. 

This system is adaptive, allowing changes in the UAV route. It is sufficient that the UAV sends the new *D_i_* value consistent with the new path (or even the new sensor scanning order) to implement the change for the next UAV scan pass. The system is also scalable because we can add new sensors and densify them provided that a minimum distance between sensors is kept (in this regard, see Equations (8) and (9)).

Let us formally determine the Δ*_i_* value used by the synchronization algorithm carried out onboard the GW. We distinguish two cases:

- Case *a*, when the next UAV pass is contiguous with the current one;
- Case *b*, when the next UAV pass is carried out after an interruption due to the refueling or other reasons.

We consider *T_s_*_,*i*_ as the transmission time set for the *i*-th sensor (for a generic transmission after the first one) according to the estimation of UAV speed and knowing the sensor coordinates and thus the corresponding distance from the origin of the UAV path. As for the *T*_0*s*,*i*_ value set in the sensor at the sensor node deployment, we assume a reference UAV route carried out for the first time starting at time *t*_0_ (i.e., the time when the UAV starts to cover the path) at the nominal speed *v_d_* and covering a distance *d_i_* to reach the closest point with the *i*-th sensor along the path. For the sake of simplicity, we define *T_s_*_,*i*_ and *T*_0*s*,*i*_ using *t*_0_ = 0. We also use *d_i_* as the distance from the origin along the *current route* to reach the *i*-th sensor in contrast to *d_i_*_,*next*_, representing the new distance of the *i*-th sensor from the same UAV path origin because of a *new route* followed by the UAV at the next pass (*d_i_*_,*next*_ = *d_i_* in case of no path change). We also consider *v_next_* as the expected UAV speed of the next UAV pass, which is estimated based on wind forecasts. 

In Case *a*, we denote $T_{i,residual\_path}$ as the estimated residual time to complete the path after reaching the *i*-th sensor, considering that the UAV must return to its origin (i.e., the starting point). This time is estimated onboard the UAV, knowing the current path characteristics and wind conditions when the *i*-th node is reached. In Case *b*, we also need to define the time the UAV will start the next route, *T_new_* (assuming that the UAV will have to stop between the current route and the new one). Therefore, Δ*_i_* results as:

(7) $${∆}_{i}=\left\{\begin{array}{l} \left(T_{c,i}-T_{s,i}\right)+T_{i,residual\_path}+\frac{d_{i,next}}{v_{next}},\;in\;case\;a\\ T_{new}-T_{s,i}+\frac{d_{i,next}}{v_{next}},\;in\;case\;b \end{array}\right..$$

where $\delta_{i}{=T}_{c,i}-T_{s,i}$ is the synchronization error (see Figure 8).

**Figure 9:**
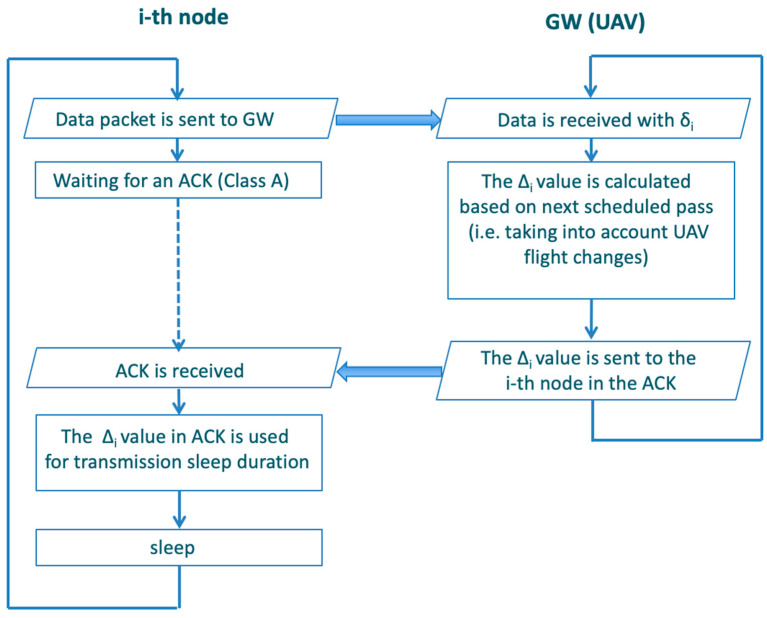
Operational steps for GW and end-node opportunistic protocol implementation.

Figure 9 depicts the operational steps followed at both the end node and the GW by the synchronization (opportunistic) protocol.

Several approaches can be implemented to cope with possible failed receptions of ACK messages that should update the timing of the next UAV pass. Here, two approaches have been proposed.

The first approach envisages the capability for the GW to include in its ACK the scheduled times for more next passes Δ*_i_* so that if the *i*-th sensor cannot communicate with the UAV in the current pass, it already knows some coarse synchronism for the next UAV passes. This approach entails a tradeoff between communication robustness and flexibility in changing the UAV’s route. Upon the next transmission, the sensor could communicate current and past measurements, thus requiring a larger payload.

The second approach envisages immediate data retransmission from the node when it does not receive the ACK within a maximum waiting window. This solution introduces a tradeoff between communication robustness and the spatial density of nodes. This is because the transmission interval needed by a node could be doubled or tripled for one or two retransmissions, thus reducing the maximum density of sensors, as will be explained in relation to the next formula (9). Finally, by enlarging the data transmission time, there is a larger probability that the sensor’s transmission will not be completed before the UAV coverage is lost.

**Figure 10:**
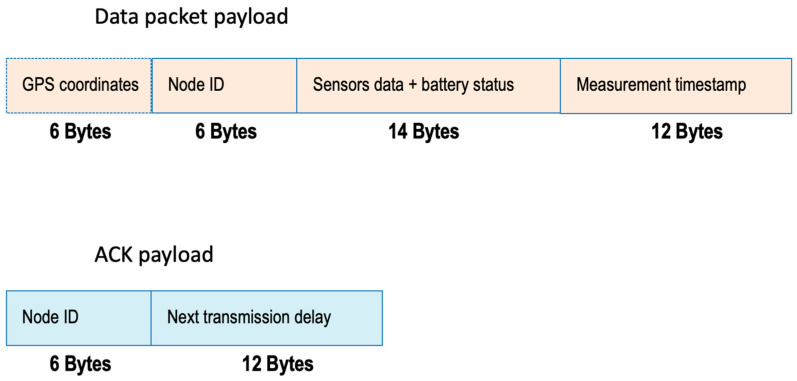
Payload for the data packet and the ACK message.

Let $T_{p}$(*L_p_*, *SF*) [$T_{A}$(*L_A_*, *SF*)] denote the transmission time of the packet (ACK) sent by a sensor node (GW) with a payload of *L_p_* (*L_A_*) bytes and using spreading factor *SF*. To determine $T_{p}$(*L_p_*, *SF*) and $T_{A}$(*L_A_*, *SF*), we need to make assumptions on the payload length for both the message sent by the sensor and the ACK sent by the GW (see Figure 10). 

Concerning the data packet, we account for 6-byte GPS coordinates (optional), a 6-byte sensor ID, 14 available bytes for collecting multiple data from the node (multiple sensors, including battery status), and a 12-byte timestamp measurement. In conclusion, the packet payload is 38 bytes long; however, it could be reduced to 22 bytes (i.e., 6 bytes for node ID, 4 bytes for sensor data, 12 bytes for timestamp) when considering a single parameter measurement with nodes georeferenced during the installation phase (thus not needing to transmit their positions). Concerning the ACK message, its payload envisages the ID of the node where this ACK and the 12-byte time specification of the delay Δ*_i_* for the next transmission are addressed, thus resulting in a total length of 18 bytes.

Based on these assumptions and using [34], the values of $T_{p}$(*L_p_*, *SF*) and $T_{A}$(*L_A_*, *SF*) are provided in Table 3 for the different *SF* values, including in the ACK case. A minimum wake-up time of 5 s, *T_w_*(*SF*), is used when it is not possible to detect an ACK in a receiver window.

Figure 8 also shows that if there is a dense sensor deployment, adjacent nodes’ coverage time intervals Δ*_vi_*(*SF*) overlap. To avoid transmission collisions, two sensors need to be separated in their transmission times *T_s_*_,*i*_ for more than the sensor message transmission time $T_{p}$(*L_p_*, *SF*) plus the time to receive the ACK message from the GW, including the receiver delay window(s), $T_{receiving\;phase}$. We have:

(8) $$T_{s,i+1}-T_{s,i}>T_{p}(L,\;\mathrm{SF})+T_{receiving\;phase}\left(SF\right),$$

where *T_receiving phase_* in the worst case can be expressed as [35,36]:

(9) $$T_{receiving\;phase}\left(SF\right)=T_{receiver\;delay1}+T_{w}(\mathrm{SF})+T_{receiver\;delay2}+T_{A}\left(L_{A}\;SF\right)$$

having $T_{receiver\;delay1}=$ $T_{receiver\;delay2}$ = 1 s. The other values are shown in Table 3. For instance, $T_{receiving\;phase}\left(SF=7\right)$ = 2077 ms.

Let us denote $T_{tot}(L,SF){=T}_{p}(L,SF)+T_{receiving\;phase}(SF)$. Based on (8) and (9), we can determine the minimum distance *d_min_* among sensors along the path to avoid their transmissions colliding because of the imposed transmission synchronism. If the sensors are not exactly on the UAV’s path, *d_min_* is considered in relation to the distance of the projections of the sensor node positions along the UAV path. We have:

(10) $$d_{min}\geq max\left\{v\left(v_{d},wind\right)\right\}\left[T_{tot}(L,SF)\right],$$

where $v\left(v_{d},wind\right)$ denotes the actual UAV speed depending on the reference speed *v_d_*, the wind conditions, and the sensor position along the path. We need to take the maximum UAV speed at sensor node’s position to consider the worst-case wind conditions.

**Figure 11:**
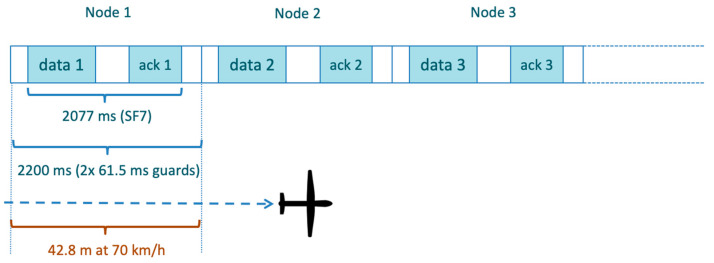
Time and spatial allocations for data sensor transmission and ACK reception (*SF* = 7).

Figure 11 shows the time durations of sending data and the ACK reception (one communication session for each node), also providing the minimum distance *d_min_* = 42.8 m when the UAV flies at 70 km/h for *SF* = 7.

**Figure 12:**
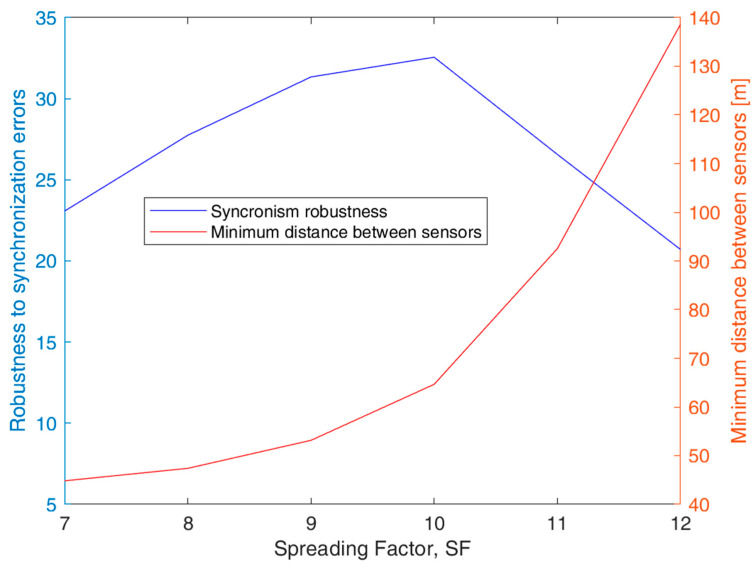
Effect of the *SF* value on the synchronism robustness η(*SF*) for *P_t_* = 6 dBm and the minimum distance between sensors *d_min_* for the reference UAV speed *v_d_* = 70 km/h.

If the *d_min_* constraint is met in the sensor deployment on the ground, there are no collisions among sensor transmissions, thus increasing system efficiency; the results for *d_min_* are shown in Figure 12, together with a parameter η(SF) describing the robustness of the synchronization scheme for ground IoT nodes-to-UAV transmissions, defined as

(11) $$\eta (SF)=\frac{\Delta_{v}(SF)}{T_{tot}(L,SF)},$$

where $\Delta_{v}(SF)$ is determined according to (1) and (5) with *P_t_* = 6 dB and $T_{tot}(L,SF)$ specified in relation to (10). We can motivate the use of parameter η(SF) in the following way: the synchronism will be more stringent if η(SF) becomes smaller. If *SF* increases, Δ*_v_*(*SF*) increases, $T_{tot}(L,SF)$ are becomes larger. The optimal condition for the synchronization protocol is when η is at its maximum.

The results in Figure 12 show that the best synchronism robustness *η* is achieved for *SF* = 10; this optimal condition is obtained at the expense of a larger *d_min_* minimum distance between sensors with respect to the case with *SF* = 7. Nevertheless, we can consider that *SF* = 10 is the optimal choice for the design of our system. Further proof of the selection of *SF* = 10 will be provided in the next section dealing with the analysis of opportunistic connectivity.

**Figure 13:**
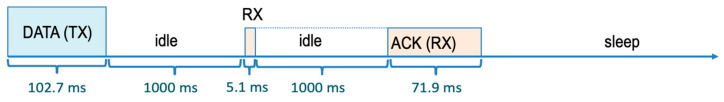
Class A communication timings for *SF* = 7 with indication of the different current conditions for the transceiver.

As for energy consumption with the synchronization protocol, the power consumption for the LoRa transceiver can be characterized by the current intensity levels in the different states (i.e., transmit, receive, idle, and sleep) and the duration of such states [32], as depicted in Figure 13. Based on the work in [7], where the power consumption of the LoRa transceiver is detailed, it is possible to obtain a duration of about 20 years with a common 2000 mAh battery with *SF* = 7, *P_t_* = 14 dBm and one packet transmission per hour with a packet size between 25 and 50 bytes. For more details and more cases, the interested reader may refer to [7].

## 5. Analysis of the Communication Opportunity between an IoT Node and the UAV

This section analyzes the synchronization issues in terms of the probability that the UAV correctly receives a measure sent by a node on the ground. The reference transmission time (with nominal UAV speed *v_d_* and nominal UAV departure time *t*_0_ = 0) for the generic *i*-th sensor is *T*_0*s*,*i*_ = $\frac{d_{i}}{v_{d}}$. Considering that the UAV has an actual speed *v* that depends on the wind conditions, the *i*-th sensor is covered by the UAV in the following time interval of duration *D_vi_* from $\frac{d_{i}-K(SF)}{v}+t_{s}$ to $\frac{d_{i}+K(SF)}{v}+t_{s}$, where $t_{s}$ denotes the time error in the UAV’s launch. In relation to (1), we have adopted the following notation: *K*(*SF*) = $\sqrt{{R(SF)}^{2}-x^{2}}$. We model time *t_s_* according to a zero-mean Gaussian random variable with standard deviation s_s_; let $N(0,\sigma_{s})$ denote the corresponding probability density function. For numerical evaluations, we consider *s_s_* = 20 s. Moreover, the UAV speed *v* is modeled according to the density function *f_v_*(*v*) in (2).

The *i*-th sensor node transmits successfully if $T_{0s,i}+T_{tot}$(*L*, *SF*) is within the interval of duration *D_vi_*. Then, suppose the transmissions of the sensors are kept at times *T*_0*s*,*i*_ without alterations to take the actual UAV speed into account. In that case, transmissions are successful according to probability *P_s_*(*d_i_*, *SF*), characterized as follows:

(12) $$\begin{array}{ll} P_{s}\left(d_{i},SF\right)=Prob. & \{\frac{d_{i}-K(SF)}{v}+t_{s}\leq T_{0s,i}\leq \frac{d_{i}+K\left(SF\right)}{v}+t_{s},\\ & {T_{0s,i}+T}_{tot}(L,SF)\leq \frac{d_{i}+K\left(SF\right)}{v}+t_{s}\}. \end{array}$$

We have two conditions in (12) that characterize the “event visibility” *V_i_* of the *i*-th node: the first is that the starting transmission time is within the UAV visibility time; the second is that the visibility time also includes the time to receive the ACK. Elaborating on the conditions in (12), we have:

(13) $$\begin{array}{cc} P_{s}\left(d_{i},SF\right)=Prob. & \{\frac{d_{i}-K(SF)}{v}-\frac{d_{i}}{v_{d}}\leq t_{s}\leq \frac{d_{i}+K\left(SF\right)}{v}-\frac{d_{i}}{v_{d}},t_{s}\\ \leq & \frac{d_{i}+K\left(SF\right)}{v}-\frac{d_{i}}{v_{d}}-T_{tot}\left(L,SF\right)\}. \end{array}$$

With our numerical settings, it is possible to prove that we have

(14) $$\begin{array}{ll} \frac{d_{i}-K(SF)}{v}-\frac{d_{i}}{v_{d}} & <\frac{d_{i}+K\left(SF\right)}{v}-\frac{d_{i}}{v_{d}}-T_{tot}\left(L,SF\right)<\frac{d_{i}+K\left(SF\right)}{v}-\frac{d_{i}}{v_{d}}\Leftrightarrow \frac{2K\left(SF\right)}{v}\\ & \geq T_{tot}\left(L,SF\right). \end{array}$$

Then, based on (14), the condition that characterizes the probability in (13) can be simplified as follows:

(15) $$P_{s}\left(d_{i},SF\right)=Prob.\left\{\frac{d_{i}-K(SF)}{v}-\frac{d_{i}}{v_{d}}\leq t_{s}\leq \frac{d_{i}+K\left(SF\right)}{v}-\frac{d_{i}}{v_{d}}-T_{tot}\left(L,SF\right)\right\}.$$

To determine (15), we condition on the *v* value and remove the conditioning using the probability density function *f_v_*(*v*) in (2):

(16) $$\begin{array}{ll} P_{s}\left(d_{i},SF\right) & =\int_{v_{min}}^{v_{max}}Prob.\{\frac{d_{i}-K(SF)}{v}-\frac{d_{i}}{v_{d}}\leq t_{s}\\ & \leq \frac{d_{i}+K\left(SF\right)}{v}-\frac{d_{i}}{v_{d}}-T_{tot}\left(L,SF\right)|v\}f_{v}\left(v\right)dv. \end{array}$$

Through some elaborations, we obtain the following result:

(17) $$\begin{array}{ll} P_{s}\left(d_{i},SF\right) & =\frac{0.59}{2}erf\left(\frac{\frac{K\left(SF\right)}{v_{d}}-T_{tot}\left(L,SF\right)}{\sqrt{2}\sigma_{s}}\right)+\frac{0.59}{2}erf\left(\frac{\frac{K\left(SF\right)}{v_{d}}}{\sqrt{2}\sigma_{s}}\right)\\ & +\frac{0.01025}{2}\int_{v_{min}}^{v_{max}}\{erf\left(\frac{\frac{d_{i}+K\left(SF\right)}{v}-\frac{d_{i}}{v_{d}}}{\sqrt{2}\sigma_{s}}\right)\\ & -erf\left(\frac{\frac{d_{i}-K\left(SF\right)}{v}-\frac{d_{i}}{v_{d}}}{\sqrt{2}\sigma_{s}}\right)\}dv. \end{array}$$

We consider that this probability decorrelates at multiple passes of the UAV close to the *i*-th sensor. Hence, if we have two subsequent passes of the UAV close to the *i*-th node (see Figure 4), the probability of communicating correctly with the *i*-th sensor in at least one of the two passes *P_s_*_,*2passes*_(*d_i_*, *SF*) can be expressed as:

(18) $$P_{s2passes}\left(d_{i},SF\right)=1-{\left(1-P_{s}\left(d_{i},SF\right)\right)}^{2}.$$

**Figure 14:**
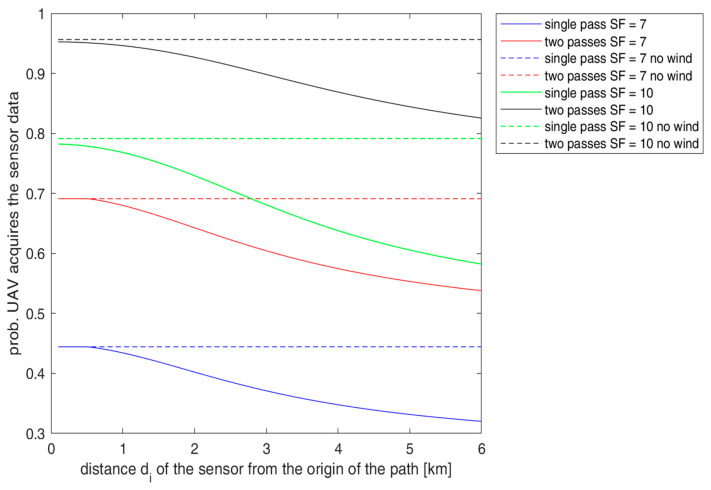
$P_{s}\left(d_{i},SF\right)$ and $P_{s2passes}\left(d_{i},SF\right)$ for *P_t_* = 6 dB, *x* = 5 m, and *SF* values of 7 and 10 as a function of the distance *d_i_* of the sensors along the UAV path.

Figure 14 provides the analytical results for $P_{s}\left(d_{i},SF\right)$ and $P_{s2passes}\left(d_{i},SF\right)$ numerically determined for *P_t_* = 6 dB, *x* = 5 m, and *SF* values of 7 and 10 as functions of the distance *d_i_* of the sensor node along the path. This graph also contains the results in the case of no wind (the ideal situation with *v* = *v_d_*) when (17) is simplified as follows:

(19) $$\begin{array}{cc} {\left.P_{s}\left(d_{i},SF\right)\right|}_{nowind}=\frac{1}{2}erf\left(\frac{\frac{K\left(SF\right)}{v_{d}}-T_{tot}\left(L,SF\right)}{\sqrt{2}\sigma_{s}}\right) & +\frac{1}{2}erf\left(\frac{\frac{K\left(SF\right)}{v_{d}}}{\sqrt{2}\sigma_{s}}\right) \end{array}$$

The value of ${\left.P_{s}\left(d_{i},SF\right)\right|}_{nowind}$ (19) is also representative of the case when the transmission times of the sensors are resynchronized during a UAV pass for the next one, based on the new expected UAV route and the wind forecasts using time *D_i_*. As noted, using the robustness parameter η(SF) in Section 4, Figure 14 shows that *SF* = 10 provides better results than *SF* = 7. In particular, with *SF* = 10, the probability of success in a single pass $P_{s}\left(d_{i},SF\right)$ is 77% at 1 km and decreases to 60% at 5 km. However, with two passes, we obtain much better results with a 94% probability of success at 1 km and an 85% probability of success at 5 km. The results improve and are close to 96% at any distance *d_i_* if we consider two UAV passes without winds or using our synchronization method. These results motivate the adoption of our proposed synchronization protocol.

In our study, we can determine *P_LoS_* at the transmission time of the generic sensor at distance *d_i_* along the UAV’s path. In our model, the nominal transmission time for the sensor at distance *d_i_* along the UAV’s route is set to time $T_{0s,i}$, assuming that the UAV ideally starts to scan its path at time *t*_0_ = 0. The UAV flies with actual speed *v* and a departure time offset from time *t*_0_ = 0 equal to *t_s_*. Then, the distance covered by the UAV along its route at the sensor transmission time is *y_UAV_*($T_{0s,i}$), calculated as:

(20) $$y_{UAV}\left(T_{0s,i}\right)=v\left(T_{0s,i}+t_{s}\right).$$

Based on our assumptions, the generic *i*-th sensor transmits when the UAV is at a distance $y_{UAV}\left(T_{0s,i}\right)-d_{i}$ with respect to the UAV’s nominal position *d_i_*. Since this sensor is at a distance *x* from the path and the UAV flies at an altitude *H*, we can determine the elevation angle $\vartheta \left(d_{i}\right)$ as follows:

(21) $$\vartheta \left(d_{i}\right)=arctan\left[\frac{H}{\sqrt{x^{2}+{\left(y_{UAV}\left(T_{0s,i}\right)-d_{i}\right)}^{2}}}\right],$$

where we consider $\vartheta \left(d_{i}\right)$ expressed in degrees.

Because of our model, $\vartheta \left(d_{i}\right)$ depends on random variables *v* and *t_s_*. Therefore, if we apply (6) with $\vartheta =\vartheta \left(d_{i}\right)$ given by (21) to express probability *P_LoS_*, this is conditioned on both *v* and *t_s_*. We remove the conditioning using a double integral and referring to those configurations of ${\left(v,t_{s}\right)}_{i}$ that allow successful a sensor to transmit when the UAV passes close to it. These configurations of ${\left(v,t_{s}\right)}_{i}$ correspond to the event *V_i_* that occurs with probability $P_{s}\left(d_{i},SF\right)$ in (17). We obtain the following result:

(22) $$P_{LoS}\left(d_{i},SF|V_{i}\right)=\frac{1}{P_{s}\left(d_{i},SF\right)}\iint_{V_{i}}^{}P_{LoS}\left(\vartheta \left(d_{i}\right)\right)f_{v}\left(v\right)N\left(0,\sigma_{s}\right)dvdt_{s}.$$

**Figure 15:**
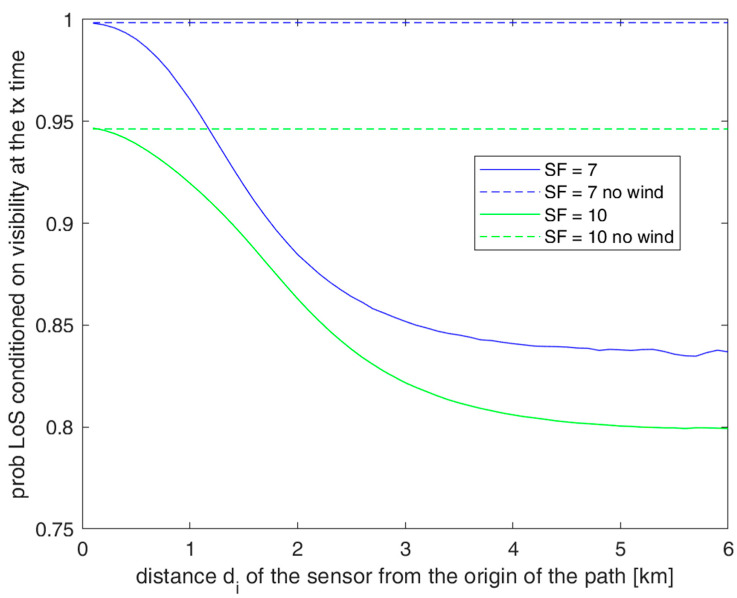
$P_{LoS}\left(d_{i},SF|V_{i}\right)$ for *P_t_* = 6 dB, *x* = 5 m, and *SF* values of 7 and 10 as a function of the distance *d_i_* of the sensors along the UAV path.

The behavior of $P_{LoS}\left(d_{i},SF|V_{i}\right)$ is shown in Figure 15 for *SF* = 7 and 10. This graph also shows the results in the no wind case that also corresponds to the situation when the sensor’s transmission is synchronized with the UAV. As performed by our protocol, we can see that the synchronization scheme permits the sensors to transmit in the best LoS conditions to the UAV. For this type of result, *SF* = 7 is better than *SF* = 10 since at *SF* = 7, the visibility interval and thus the range of $\vartheta \left(d_{i}\right)$ values have a smaller extension around the optimal central value. On the other hand, we have considered here conditioned probabilities $P_{LoS}\left(d_{i},SF|V\right)$ to the UAV in the visibility interval so that, by multiplying $P_{LoS}\left(d_{i},SF|V_{i}\right)$ by $P_{s}\left(d_{i},SF\right)$ to obtain the joint probability of transmission during the interval of UAV visibility and LoS conditions, we have that *SF* = 10 is the most convenient choice.

## 6. Experiments and Results

This section reports the results of the propagation study on the use of LoRa technology in a rural scenario, as well as the validation of the opportunistic protocol obtained through tests carried out in the laboratory, which are focused on the correct functionality of the synchronization protocol between the GW and the nodes.

### 6.1. Propagation Study

This sub-section describes an experimental testbed for a preliminary study on ground propagation conditions. Our hardware is based on the Semtech SX1276 LoRa transceiver embedded on both the GW and the end node, operating at 868 MHz (EU frequency band for LoRa). Nodes are equipped with an Arduino UNO board and powered by a 9 V battery. The GW adopts a 400 MHz ar9331 processor with 64 MB RAM and 16 MB flash memory for the GW management system and an ATMega328P MCU with 2 kB of RAM and 32 kB of flash memory for the LoRa part. The LoRa communication is set with an uplink transmission power *P_t_* = 25 mW (i.e., 14 dBm). 

In this case, we study propagation, and we do not impose special constraints on *P_t_* because we like to use a strong signal. At both the end node and the GW, antennas are normal-mode helical antennas (with a radiation pattern similar to a 1/4−λ monopole antenna) used in the vertical position. Hence, we consider the following antenna gains at both transmitter and receiver: *G_tx_* = *G_rx_* = 2 dBi. Moreover, we have a total cable and connector losses *L_con_* = 2 dB. The spreading factor is set to 7, with a bandwidth of 125 kHz, coding rate = 4/5, payload = 8 bytes, and preamble length = 8 bytes (sensor ID plus two additional bytes). All the setup parameters have been summarized in Table 4.

**Figure 16:**
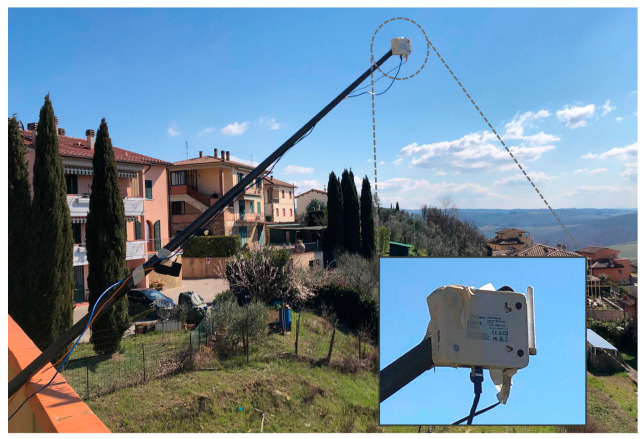
LoRa gateway.

**Figure 17:**
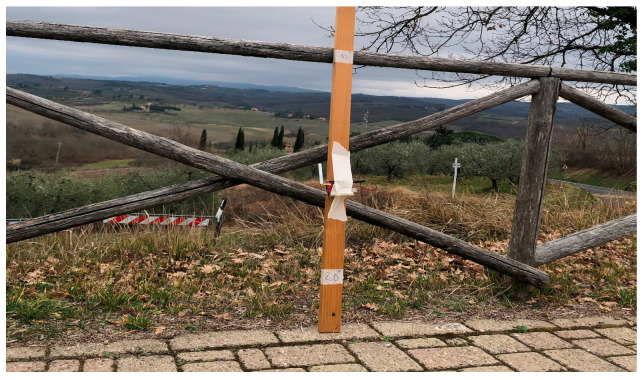
LoRa sensor nodes at different altitudes from the ground.

In this work, we have conducted a preliminary study focused on the propagation of UAV adoption and emulating its effects, so the GW has been placed on top of a hill, as detailed below. The GW has been installed in a fixed location, while sensor nodes have been disseminated in different positions in the territory. From this study, we expect to obtain a useful benchmark to evaluate the path loss when sensors are very close to the ground. Figure 16 shows the GW, attached 2.5 m outside a building, at 10 m from the ground. The end nodes have been placed in LoS positions at different distances from the GW (i.e., 0.5, 1, 1.5, and 3 km, obtained by GPS coordinates). The sensor node has been positioned at five different heights from the soil (i.e., 0, 20, 50, 100, and 150 cm), as depicted in Figure 17.

**Figure 18:**
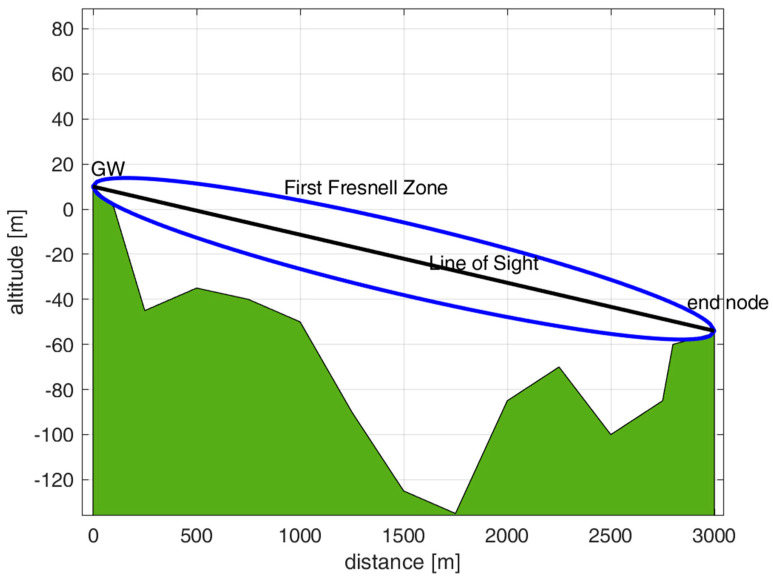
Representation of the testbed location with the altimetric profile. This graph also shows the FFZ (in blue color) and the LoS (in black color) between the GW and end node placed at a distance of 3 km. The effects of the ‘spherical’ Earth’s surface are not visible at these small distances. The FFZ maximum clearance is about 16 m under these conditions.

The selected distances for our study allow us to consider the propagation in far-field conditions. Figure 18 provides the altitude profile of the deployment testbed for checking the First Fresnel Zone (FFZ) clearance condition. This zone is not obstructed because the ellipsoid from the transmitter and receiver in Figure 18 is free from obstacles or is obstructed by less than 20%. Hence, the scenario is in LoS conditions. The maximum clearance distance of FFZ, *F*_1_, can be determined according to the following formula:

(23) $$F_{1}=8.656\sqrt{\frac{d}{1000f\left[GHz\right]}}\left[m\right],$$

where *f* is the transmission frequency in GHz (0.868 in the LoRa case for the EU zone) and *d* is the distance between the transmitter and receiver in m. For instance, *F*_1_ = 9.3 m for *d* = 1 km and *F*_1_ = 16 m for *d* = 3 km.

For the sake of completeness, we have to consider that propagation experiments carried out in the presence of flat ground (this is not our case, as shown in the following Figure 18, where the GW is on top of a hill and the valley does not have a constant profile) are characterized by a two-ray propagation model with a cut-off distance *d_t_*. Before such a distance, the signal reflections positively or negatively sum so that the path loss attenuation oscillates even for slight differences in the distances; above such a cut-off distance, the path loss attenuation becomes more regular (with an exponent g = 4). We have:

(24) $$d_{t}=\frac{4h_{n}H}{\lambda }\left[m\right],$$

where *h_n_* is the altitude of the sensor node from the ground, l is the wavelength and *H* is the UAV’s altitude. For instance, for *h_n_* = 0.2 m and *H* = 120 m, *d_t_* = 278 m for the 868 MHz EU LoRa case.

Our experiments have been conducted in sunny weather. Further studies on the impact of different weather conditions and vegetation on LoRa propagation are beyond the scope of this work.

We study a path loss model as in (3) by introducing a log-normal shadowing term X. In particular, X is a Gaussian random variable with zero-mean and standard deviation s_p_. This model is based on three parameters as *L*(*d*_0_), g, and s_p_. With respect to (3), we neglect the attenuation due to vegetation in this scenario and refer to far-field conditions. Extra complexity in the propagation study is due to the closeness of sensors to the ground that may partially obstruct the FFZ for the transmission with the GW [36], as further discussed below.

For each distance (i.e., 0.5, 1, 1.5, 3 km) of the sensors from the GW, the Received Signal Strength Indicator (RSSI) and the Signal-to-Noise Ratio (SNR) values have been measured at the GW side. Such measurements are related to the LoRa packets transmitted by the nodes. RSSI reports the amount of power received, including the received useful signal and the background noise. Then, we calculated the received power level *P_r_* from both RSSI [dBm] and SNR [dB] measurements as follows:

(25) $$P_{r}=RSSI-10{log}_{10}\left(1+\frac{1}{10^{\frac{SNR}{10}}}\right)[dBm].$$

The *P_r_* value is used to determine the path loss attenuation *L*(*d*) according to the link budget terms as follows:

(26) $$L\left(d\right)=P_{r}-P_{t}-G_{rx}-G_{tx}-L_{con}\;[\mathrm{dB}].$$

**Figure 19:**
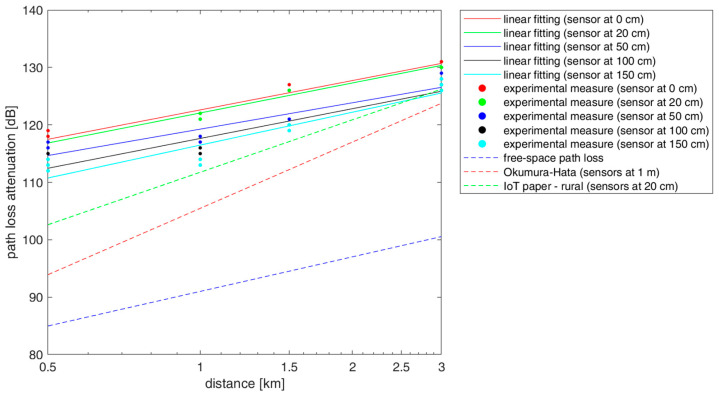
Path loss model based on measurements (circles with distinct colors depend on the distance of the sensor from the ground) and comparison with models from the literature (i.e., freespace path loss, Okumura–Hata for rural areas, and another model from [29]).

We performed multiple measurements of both the RSSI and SNR for distances of 0.5, 1, 1.5, and 3 km. We repeated the measurement of each distance five times, varying the node positions to avoid possible measurement biases due to small local obstacles. Then, we used (25) and (26) to determine the corresponding path loss attenuation *L*(*d*) values. We have adopted a linear fitting (i.e., least square) method with a “non-fixed intercept” *L*(*d*_0_) that is considered an outcome of the fitting process [37]. The slope of the fitted line using log-scale distance yields the path loss exponent g. Finally, the standard deviation of the shadowing s_p_ has been obtained as the standard deviation of the difference between the path loss measures and the corresponding linear fitting. The results of the linear fitting are provided in Figure 19, which also shows the comparison with the free-space path loss model, the Okumura–Hata model for rural areas (sensors at 1 m from the ground), and the path loss model used in Section 3 derived from [29] in a similar scenario (sensors at 20 cm from the ground). Confidence intervals (max values) are ± 0.5% around the central (mean) value, considering five repetitions and the method to compute confidence intervals based on Student’s distribution. The parameter values of our path loss model fitting the measurements are shown in Table 5. 

Based on the results shown in Figure 19 and Table 5, we can say that, in our scenario, the path loss exponent is close to γ = 2, as in the free-space case, but there are cases with a γ below 2, which could be caused by reflections off of buildings close to the experimental site and diffraction by trees [36,38,39]. We can see that this model has significant deviations with respect to other propagation models for rural areas in the literature.

Our results also show an impact on the path loss due to closeness of the sensor to the ground. In particular, the path loss increases as the sensor’s distance from the ground decreases: there is an increase of 6 dB from the sensor at 150 cm to the sensor on the ground. This is an interesting aspect to consider for this type of IoT application. The shadowing standard deviation σ_p_ is relatively low, around 1 dB for sensors closer to the ground and around 2 dB for sensors at 50, 100, and 150 cm from the ground.

Even if the proposed path loss model from our experiments adopts a ground GW positioned on top of a hill, we can consider that these results can (approximately) represent the path loss attenuation for the case with the GW onboard a UAV flying at a low altitude. As explained in the concluding sections, the study of the path loss with a GW onboard a UAV is left to future work.

### 6.2. Preliminary Validation of the Opportunistic Protocol in the Laboratory

This section describes the experimental testbed conducted in the laboratory to preliminarily validate the synchronization protocol, specifically designed for the communication between ground nodes and UAV-GW. The lab experiment envisages the adoption of sensor nodes and a moving GW (Dragino LG01N, which will be placed onboard a UAV in a real case) connected to the Internet via WiFi. This scenario has been realized in the lab by placing two nodes on a table at a mutual distance of 10 m and moving the GW at a constant speed to simulate the UAV flying over the two nodes, as detailed below. We have set *P_t_* = 0 dBm and removed the LoRa antennas mounted on both the sensor nodes and the GW, thus increasing the attenuation loss. This setup has resulted in a coverage radius *R* of about 4 m. Such coverage has been empirically verified through measurements based on RSSI values of packets transmitted from the node to the moving GW. To emulate the pass of the UAV, the GW is moved closer to and further away from the nodes at a speed *v* of about 0.2 m/s so that its visibility time *D_vi_* = 2*R*/*v* = 8/0.2 = 40 s is consistent with the UAV’s visibility time, as shown in Figure 7 for *P_t_* = 3 dBm and *SF* = 7. Our laboratory model is thus adopting a scale factor of 1:100 with respect to the size of the actual scenario. By conducting this test, we have verified the node communication timing and the correct functionality of the opportunistic protocol with the exchange of sensor packets and the GW ACK. 

**Figure 20:**
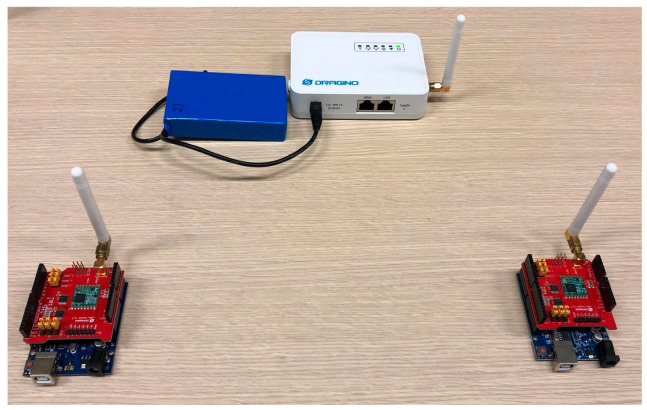
Hardware for laboratory setup with two sensor nodes and a GW (LoRa antennas have been removed when testing the opportunistic protocol in the laboratory to reduce the coverage range).

The laboratory demo hardware (sensor nodes and GW) is depicted in Figure 20 and is the same hardware adopted for the propagation study described in the previous sub-section.

For each node, the route management algorithm provides the sleeping time duration Δ*_i_* based on the synchronism adjustment term δ*_i_* in a specific function, and this value has been retrieved by the GW code. Then, the sleeping time duration Δ*_i_* is correctly delivered to each node through ACKs. The reception of such values has been verified in the laboratory using Arduino IDE’s serial monitor on the nodes. Finally, the received sleeping time values have been correctly elaborated and adopted by the nodes, which woke up at the correct time for the next transmission corresponding to the next GW pass.

In our tests, the collision events were not investigated due to the extremely low number of nodes (i.e., two nodes, out of range from each other) and the sporadic transmissions (i.e., one packet sent every two minutes). However, a specific routine can be introduced in the GW to detect packet losses (thus including the effects of collisions), which involves counting the received packets and comparing them with the expected number of packets to be received (i.e., the number of nodes to be visited).

**Figure 21:**
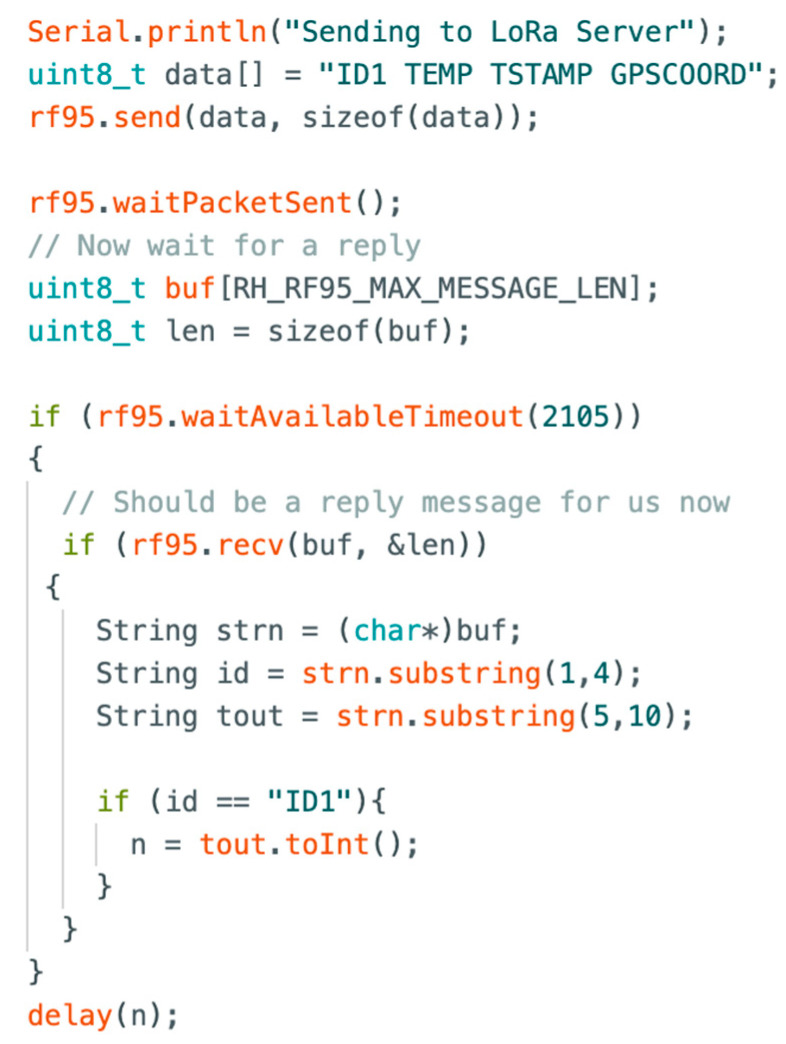
Code for the sensor node—client.

**Figure 22:**
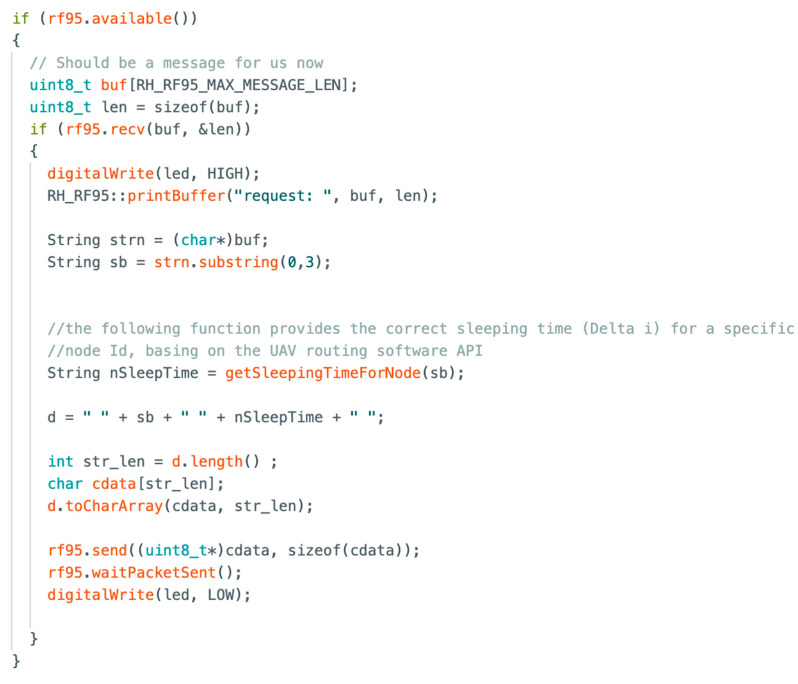
Code for the GW—server.

Figure 21 and Figure 22 show key parts of the code implemented on the GW and end nodes for the synchronization protocol, as depicted in Figure 9. Specifically, Figure 21 provides the code for the sensor node, showing that the device sends its ID and a dummy temperature measure. Then, it waits for the ACK from the GW, and when the ACK from the GW is received, it is parsed to check if the ACK is related to its data packet. Then, the sleeping time Δ*_i_* is obtained from the ACK and used to wait for the subsequent transmission attempt. Figure 22 shows the code for the GW/server node waiting for packets. When a packet is received, the server parses it to obtain the node ID, which is needed to calculate the sleeping time specific to that node (i.e., through a specific function interfaced with the UAV’s route management system, as explained in Section 4). Then, the GW sends back an ACK containing node ID and its sleeping time Δ*_i_*. It should be noticed that the parameters in the payload of the message sent by the node (i.e., ID1 TEMP TSTAMP GPSCOORD) and related ACK (i.e., ID1 5000) in Figure 21 and Figure 22 are just dummy values to validate the protocol’s behavior. 

## 7. Discussion

The work carried out in this paper represents an interesting step toward the study of the communication between ground sensors and a GW flying onboard a UAV. First of all, we have identified LoRa technology as a valid solution to allow communication from low-power sensors to the GW. Second, we have investigated propagation aspects and identified a protocol to synchronize the LoRa node transmissions with the UAV pass, showing some criticalities and preferred settings in terms of the LoRa spreading factor and the transmission power levels for the sensor nodes (i.e., *SF* = 10 and *P_t_* = 6 dB).

It is difficult to assess a propagation model that is quite general since the actual path loss is strongly influenced by the environmental conditions surrounding the transmitter and receiver and the obstacles between them. In our preliminary path loss study, we highlighted the importance of placing the GW on top of a hill to achieve better LoS with distant nodes. Signal propagation is affected by local reflections, whose impact on the received power level is more significant when the receiver and transmitter are close. Then, an important lesson learned is to investigate the path loss at a distance larger than the so-called cut-off distance to minimize these effects. Further work is also needed to replicate our experiments using a UAV to determine a path loss model for ground-to-air and air-to-ground communications. For such experiments, a rotary-wing UAV is needed to hover over a given position. 

As for limitations concerning the opportunistic protocol, we have addressed only the communication protocol between the nodes and the GW in the laboratory without using a UAV. Another limitation of the correct functionality of the system is represented by meteorological conditions, namely, strong wind and rain events, which affect the UAV’s flight capability and the LoRa communication range. In addition to this, vegetation and the presence of dense trees can reduce the visibility of the nodes and increase path loss propagation, thus requiring a site-specific propagation study.

Further development will be needed to conduct field experiments with UAVs to test the robustness of the synchronization scheme. 

Our UAV-based IoT system and the designed synchronization protocol are useful not only for smart agriculture and environmental monitoring but can also be adopted for emergency applications, infrastructure monitoring, rescue operations, and other scenarios involving opportunistic connectivity where coverage infrastructure is missing.

## 8. Conclusions

This paper studies the opportunistic communication of ground IoT nodes with a GW flying onboard a UAV for scanning rural or remote areas for smart agriculture and environmental monitoring applications. This system is conceived for low-power IoT nodes in remote areas without terrestrial connectivity.

A UAV flight dynamics model has been exploited based on the Matlab UAV Toolbox, which allows the prediction of the next time the UAV will pass close to each sensor along a pre-determined route. The LoRa/LoRaWAN radio technology has been adopted for the communication of IoT nodes with the GW because such technology allows low-power, long-range communications. 

An opportunistic protocol has been proposed to allow communication between sensor nodes and the GW on the UAV. Sensor nodes use LoRa Class A, keeping the communication module in sleep mode most of the time and waking up and transmitting data right when the UAV passes close to them. This approach allows for significantly better energy efficiency and longer endurance of the sensors’ batteries. 

Furthermore, an analytical approach based on a system model has allowed for predicting the probability that the sensor can communicate during the UAV visibility time. 

On-the-field propagation measurements have been carried out to assess how the closeness of the sensors to the ground can impact path loss attenuation. A preliminary laboratory implementation has allowed the validation of the designed synchronization protocol for ground IoT nodes. Future work is needed to replicate the propagation study and the protocol test using a GW on the UAV and ground sensors.

## Figures and Tables

**Table 1:** Main contributions in literature.

Ref.	Description	Advantages	Disadvantages
[14]	Review of energy-efficient practices and strategies for sustainable IoT	Comprehensive review of solutions	Not related to GW mobility
[15]	Investigation of current practices, challenges, and policies related to LPWAN	Discussion on threats of e-waste related to IoT data centers, sensor nodes, and machine-to-machine communications	Not related to GW mobility
[16]	Novel energy-efficient routing scheme for a smart grid environment	Improvement in energy consumption with respect to other routing protocols	Not related to GW mobility
[17]	WiFi-based IoT network configurations for soil monitoring	Propagation tests performed by adopting different vegetation conditions	Not related to GW mobility, WiFi-based
[18]	LoRa sensor network for water quality monitoring in coastal areas	Proof-of-concept tested in a real environment	Not related to GW mobility
[19]	Customized beacon sent by a moving GW to announce its reachability	Asynchronous communications	Awake transceiver node to receive ping messages
[20]	Opportunistic multi-hop routing protocol for LoRa-based intermittently connected networks	Support for multi-hop data exchange	Not using a GW on UAVs
[21]	Mobile and fixed nodes equipped with WiFi and LoRa	Management of multiple sink stations in the network	Use of WiFi (no low-power)
[22]	A secondary Wake-up Radio always scanning for devices	Asynchronous wake-up of the main node with low latency	Short-range link, limiting the UAV flying altitude
[23]	Monitoring system for a forest based on LoRa and UAV	Asynchronous communications	Sensor node always active in receiving mode

**Table 2:** Time to reach our exemplary sensor in Figure 4 after leaving the origin and the related UAV speed for the different wind conditions with *SF* = 7.

Exemplary Sensor in Figure 4	Central Time of Visibility for the Sensor, *T_c_*_,*i*_	Actual UAV Speed *v* when Passing over the Sensor
Red case for winds	175 s	54 km/h
Blue case for winds	112 s	84 km/h
Green case (no wind)	128 s	70 km/h

**Table 3:** Time on air for data packets and ACK packets for the assumed payload sizes and the different *SF* values for a code rate of 4/8 and bandwidth of 125 kHz.

	*SF* = 7	*SF* = 8	*SF* = 9	*SF* = 10	*SF* = 11	*SF* = 12
$T_{p}$(*L_p_*, *SF*)	102.7 ms	184.8 ms	328.7 ms	616.4 ms	1314.8 ms	2465.8 ms
$T_{A}$(*L_A_*, *SF*)	71.9 ms	133.6 ms	246.8 ms	452.6 ms	905.2 ms	1810.4 ms
$T_{w}$(*SF*)	5.12 ms	10.24 ms	20.48 ms	40.96 ms	81.92 ms	164 ms

**Table 4:** Setup parameters.

Parameter	Value
Transmission power	25 mW–14 dBm
Frequency	868.1 MHz
Spreading factor	7
Coding rate	4/5
Bandwidth	125 kHz
Preamble/payload lengths	8/8 bytes
Antenna gain (TX)	2 dBi
Antenna gain (RX)	2 dBi

**Table 5:** Path loss model.

Sensor Altitude from the Ground (cm)	*L*(*d*_0_), Path Loss Attenuation at 1 km (dB)	γ, Path Loss Exponent	σ_p_, Shadowing st. Deviation (dB)
0	122.6	1.7	1.1
20	122	1.8	1.2
50	119.2	1.6	2
100	117.6	1.8	2.1
150	116.4	2	2.1

## Data Availability

Data is contained within the article.

## References

[1] Mekkia K., Bajica E., Chaxela F., Meyer F. (2019). A Comparative Study of LPWAN Technologies for Large-Scale IoT Deployment. ICT Express.

[2] Andreadis A., Giambene G., Zambon R. Low-Power IoT Environmental Monitoring and Smart Agriculture for Unconnected Rural Areas. Proceedings of the 20th Mediterranean Communication and Computer Networking Conference.

[3] Sciddurlo G., Petrosino A., Quadrini M., Roseti C., Striccoli D., Zampognaro F., Luglio M., Perticaroli S., Mosca A., Lombardi F. (2022). Looking at NB-IoT over LEO Satellite Systems: Design and Evaluation of a Service-Oriented Solution. IEEE Internet Things J..

[4] Elghonaimly A.N. (2020). LoRaWAN for Air Quality Monitoring System. Master’s Thesis.

[5] CEPT (2021). ERC Recommendation 70-03. https://docdb.cept.org/download/3700.

[6] Semtech LoRa Platform for IoT. https://www.semtech.com/lora.

[7] Cheong P.S., Bergs J., Hawinkel C., Famaey J. Comparison of LoRaWAN Classes and their Power Consumption. Proceedings of the IEEE Symposium on Communications and Vehicular Technology (SCVT).

[8] Delgado-Ferro F., Navarro-Ortiz J., Chinchilla-Romero N., Ramos-Munoz J.J. (2022). A LoRaWAN Architecture for Communications in Areas without Coverage: Design and Pilot Trials. Electronics.

[9] Nemati M., Al Homssi B., Krishnan S., Park J., Loke S.W., Choi J. (2022). Non-Terrestrial Networks with UAVs: A Projection on Flying Ad-Hoc Networks. Drones.

[10] Ferrero F. HAB_Relay_STM32Contest, with Description and Supporting Material. https://github.com/FabienFerrero/HAB_Relay_STM32Contest.

[11] Giambene G., Addo E.O., Kota S. 5G Aerial Component for IoT Support in Remote Rural Areas. Proceedings of the IEEE 5G World Forum (5GWF).

[12] Karar M.E., Alotaibi F., AL Rasheed A., Reyad O. (2021). A Pilot Study of Smart Agricultural Irrigation Using Unmanned Aerial Vehicles and IoT-Based Cloud System. Int. J. Inf. Sci. Lett..

[13] Di Franco C., Buttazzo G. (2016). Coverage Path Planning for UAVs Photogrammetry with Energy and Resolution Constraints. J. Intell. Robot. Syst..

[14] Alsharif M.H., Jahid A., Kelechi A.H., Kannadasan R. (2023). Green IoT: A Review and Future Research Directions. Symmetry.

[15] Albreem M.A., Sheikh A.M., Bashir M.J.K., El-Saleh A.A. (2023). Towards green Internet of Things (IoT) for a sustainable future in Gulf Cooperation Council countries: Current practices, challenges and future prospective. Wirel. Netw..

[16] Repuri R.K., Darsy J.P. (2023). Energy-Efficient LoRa Routing for Smart Grids. Sensors.

[17] García L., Parra L., Jimenez J.M., Parra M., Lloret J., Mauri P.V., Lorenz P. (2021). Deployment Strategies of Soil Monitoring WSN for Precision Agriculture Irrigation Scheduling in Rural Areas. Sensors.

[18] Sendra S., Parra L., Jimenez J.M., García L., Lloret J. (2022). LoRa-based Network for Water Quality Monitoring in Coastal Areas. Mob. Netw. Appl..

[19] Ferreira A.E., Ortiz F.M., Costa L.H.M.K., Foubert B., Amadou I., Mitton N. (2020). A Study of the LoRa Signal Propagation in Forest, Urban, and Suburban Environments. Ann. Telecommun..

[20] Le Sommer N., Touseau L. LoRaOpp: A Protocol for Opportunistic Networking and Computing in LoRa Networks. Proceedings of the 18th International Conference on Wireless and Mobile Computing, Networking and Communications (WiMob 2022).

[21] Almeida R., Oliveira R., Sousa D., Luis M., Senna C., Sargento S. A Multi-Technology Opportunistic Platform for Environmental Data Gathering on Smart Cities. Proceedings of the 2017 IEEE Globecom Workshops.

[22] Piyare R., Murphy A.L., Kiraly C., Tosato P., Brunelli D. (2017). Ultra Low Power Wake-Up Radios: A Hardware and Networking Survey. IEEE Commun. Surv. Tutor..

[23] Park S., Yun S., Kim H. Forestry Monitoring System using LoRa and Drone. Proceedings of the 8th International Conference on Web Intelligence, Mining and Semantics.

[24] Global Wind Atlas. https://globalwindatlas.info/.

[25] MATLAB UAV Toolbox. https://it.mathworks.com/help/uav/getstarted.html.

[26] MATLAB. https://it.mathworks.com/products/matlab.html.

[27] Kendoul F., Yu Z., Nonami K. Embedded Autopilot for Accurate Waypoint Navigation and Trajectory Tracking: Application to Miniature Rotorcraft UAVs. Proceedings of the IEEE International Conference on Robotics and Automation.

[28] Khawaja W., Guvenc I., Matolak D.W., Fiebig U.C., Schneckenburger N. (2019). A Survey of Air-to-Ground Propagation Channel Modeling for Unmanned Aerial Vehicles. IEEE Commun. Surv. Tutor..

[29] El Chall R., Lahoud S., El Helou M. (2019). LoRaWAN Network: Radio Propagation Models and Performance Evaluation in Various Environments in Lebanon. IEEE Internet Things J..

[30] ITU-R (2021). Attenuation in Vegetation.

[31] Drone Laws: Regulations by State and Country. https://uavcoach.com/dronelawsinitaly/.

[32] Wireless, Sensing & Timing; SX1276/77/78/79; Semtech Corporation: Camarillo, CA, USA, 4 March 2015. https://cdnshop.adafruit.com/productfiles/3179/sx1276_77_78_79.pdf.

[33] AlHourani A., Kandeepan S., Lardner S. (2014). Optimal LAP Altitude for Maximum Coverage. IEEE Lett. Wirel. Commun..

[34] LoRa Airtime Calculator. https://avbentem.github.io/airtime-calculator/ttn/eu868/30.

[35] ITU-R Propagation Data and Prediction Methods for the Planning of Short Range Outdoor Radiocommunication Systems and Radio Local Area Networks in the Frequency Range 300 MHz to 100 GHz; Recommendation P.14111; 2005. https://www.itu.int/rec/R-REC-P.1411/en.

[36] Klaina H., Vazquez Alejos A., Aghzout O., Falcone F. (2018). Narrowband Characterization of Near-Ground Radio Channel for Wireless Sensors Networks at 5G-IoT Bands. Sensors.

[37] Capuzzo M. (2020). Reliable LoRaWAN Links: Performance Analysis. Master’s Thesis.

[38] Bianco G.M., Giuliano R., Marrocco G., Mazzenga F., Mejia-Aguilar A. (2021). LoRa System for Search and Rescue: Path Loss Models and Procedures in Mountain Scenarios. IEEE Internet Things J..

[39] Wojcicki P., Zientarski T., Charytanowicz M., Lukasik E. (2021). Estimation of the Path-Loss Exponent by Bayesian Filtering Method. Sensors.

